# How nitric oxide helps update memories

**DOI:** 10.7554/eLife.53832

**Published:** 2020-01-08

**Authors:** Daniel JE Green, Andrew C Lin

**Affiliations:** Department of Biomedical ScienceUniversity of SheffieldSheffieldUnited Kingdom

**Keywords:** associative learning, memory dynamics, dopamine, cotransmitter, nitric oxide, mushroom body, *D. melanogaster*

## Abstract

Some dopaminergic neurons release both dopamine and nitric oxide to increase the flexibility of olfactory memories.

**Related research article** Aso Y, Ray RP, Long X, Bushey D, Cichewicz K, Ngo TT, Sharp B, Christoforou C, Hu A, Lemire AL, Tillberg P, Hirsh J, Litwin-Kumar A, Rubin GM. 2019. Nitric oxide acts as a cotransmitter in a subset of dopaminergic neurons to diversify memory dynamics. *eLife* **8**:e49257. doi: 10.7554/eLife.49257

To find food and avoid danger in changing environments, animals need to be able to learn to associate specific sensory stimuli, such as odors, with reward or punishment. Such memories must also adapt to reflect new circumstances. How is such memory flexibility achieved?

An excellent model system for studying these questions is the fruit fly *Drosophila melanogaster*, which can learn to associate certain odors with reward or punishment. Odor-associated memories are formed in a part of the fly brain called the mushroom body, which contains about 2000 neurons called Kenyon cells, which are connected to other neurons called mushroom body output neurons (MBONs; Amin and Lin, 2019). A small percentage of Kenyon cells (~5–10%) is activated by a given odor, which triggers a behavioral response (via the MBONs), such as approaching the odor or avoiding it.

The connections between the Kenyon cells and the MBONs can be altered by subpopulations of dopaminergic neurons that carry reward or punishment information (Hige et al., 2015). When a fly smells an odor at the same time as it experiences reward or punishment, certain dopaminergic neurons are activated at the same time as an odor-specific set of Kenyon cells. This simultaneous activation alters Kenyon cell-to-MBON connections, leading to an altered odor-induced behavior (positive or negative memory).

Now, in eLife, Gerald Rubin and colleagues at Janelia Research Campus, Columbia University, and the University of Virginia – including Yoshinori Aso as first author – report that some dopaminergic neurons release nitric oxide as well as dopamine, and that this nitric oxide acts in the ‘opposite direction’ to the dopamine, and also on a slower timescale (Aso et al., 2019). These opposing signals ensure that odor-associated memories are forgotten after time passes, allowing for memory flexibility.

Different types of dopaminergic neurons innervate different ‘compartments’ in the mushroom body (Figure 1A). Previous work showed that activating a dopaminergic neuron that responds to punishment (reward) while the fly smells an odor can induce a negative (positive) artificial memory, so the fly later avoids (approaches) that particular odor (Aso and Rubin, 2016; Aso et al., 2012; Aso et al., 2010). This memory can form and decay quickly or slowly depending on what dopaminergic neuron induced it.

**Figure 1.**
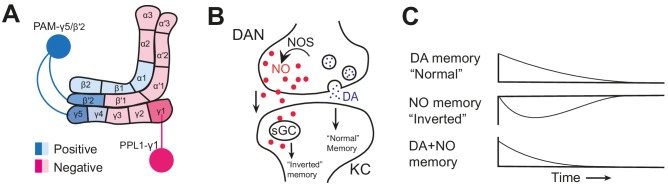
Nitric oxide signaling in the mushroom body. (**A**) Schematic diagram of the *Drosophila melanogaster* mushroom body showing the compartments innervated by dopaminergic neurons (DANs) that induce positive memories (light/dark blue) and negative memories (light/dark pink), and the neurons studied by Aso et al.: PAM-γ5/PAM-β’2a (dark blue) and PPL1-γ1pedc (dark pink). (**B**) Nitric oxide synthase (NOS) generates nitric oxide (NO, red dots) in a dopaminergic neuron (DAN, top). NO diffuses into the Kenyon cell (KC, bottom), where it binds soluble guanylate cyclase (sGC) to produce an ‘inverted’ memory. At the same time, dopamine (DA, blue dots) is released from the dopaminergic neuron via synaptic vesicles, creating a ‘normal’ memory. (**C**) NO-induced memories (middle) have the opposite valence to dopamine-induced memories (top) and are slower to both form and decay. Dopamine and NO interact (bottom) to produce memories that form and decay quickly and are easily updated.

One would expect that if a fly cannot produce dopamine (because an enzyme needed to make it is knocked out), these memories would not form. However, Aso et al. encountered something surprising when they performed experiments on flies that lacked dopamine: activating a dopaminergic neuron called PPL1-γ1pedc (which normally induces negative memories) induced a positive memory, while activating PAM-γ5/PAM-β’2a (which normally induce positive memories) induced a negative memory. These 'inverted memory' results are consistent with previous results that dopamine-less flies form positive memories when they are trained to associate an odor to a negative experience like an electric shock (Riemensperger et al., 2011). Aso et al. went further by pinning down this inverted memory to specific neurons. Thus, dopaminergic neurons can induce memory formation without dopamine, suggesting that they might use another neurotransmitter as a co-transmitter.

But what co-transmitter? Using RNA-seq, Aso et al. looked at gene expression in different dopaminergic neuron subtypes to see what the subtypes inducing inverted memories express that others do not. This analysis pointed to nitric oxide synthase, the enzyme that produces nitric oxide (NO). This gas has several roles in the body, including acting as a neurotransmitter in mammalian long term memory (Harooni et al., 2009). Moreover, the major downstream signaling molecule for NO, soluble guanylate cyclase, is expressed in Kenyon cells, suggesting that NO might diffuse across cell membranes from dopaminergic neurons to Kenyon cells (Figure 1B).

Aso et al. showed that both NO production in dopaminergic neurons and soluble guanylate cyclase signaling in Kenyon cells are required for inverted memory. When either is disrupted, activating the dopaminergic neurons described above in dopamine-less flies no longer produces inverted memories. However, when dopamine levels were restored in flies lacking both NO and dopamine, normal memory formation took place, indicating NO is not required for normal memory (as opposed to inverted memory).

Why would these dopaminergic neurons simultaneously induce two opposite memories? The inverted memory induced by NO forms more slowly than the normal memory induced by dopamine, suggesting that the inverted memory could cancel out the normal memory after some time passes (Figure 1C). Consistent with this hypothesis, normal memories in NO-deficient flies last much longer than in wild-type flies, suggesting that the inverted memory is actually a kind of active forgetting. Indeed, NO-deficient flies struggle to update memories based on new information.

Aso et al. used computational modeling to understand how the dopamine-induced normal memory and the NO-induced inverted memory interact with each other. The results suggest that dopamine and NO could modulate three classical parameters of synaptic strength (quantal size, release probability, and number of release sites), which give the mean postsynaptic response when multiplied together. It will be interesting to test which, if any, of these parameters are altered during learning.

The mushroom body is architecturally similar to the vertebrate cerebellum (Farris, 2011), and this study highlights further molecular parallels. In the cerebellum, NO can induce a change in the connection between granule cells (akin to Kenyon cells) and Purkinje cells (akin to MBONs) in the opposite direction to the change that happens during normal learning (Lev-Ram et al., 2002). Whether due to conservation from an ancestral brain structure or convergent evolution to a useful architecture, these parallels underscore the usefulness of the mushroom body as a model system to understand memory dynamics. It will be interesting in the future to explore how opposing co-transmitters modulate memory timescales in other systems, and whether such systems are involved in human learning disabilities or dementia.
